# Perceived anxiety and depression and associated factors among women inmates with a long-term sentence in Thailand

**DOI:** 10.1371/journal.pone.0299318

**Published:** 2024-03-01

**Authors:** Malee Sunpuwan, Suchada Thaweesit, Kanchana Tangchonlatip

**Affiliations:** Institute for Population and Social Research, Mahidol University, Nakhon Pathom, Thailand; FIOCRUZ: Fundacao Oswaldo Cruz, BRAZIL

## Abstract

**Background:**

In Thailand, the growing prevalence of mental health problems among the increasing number of adult female prisoners has emerged as a significant public health concern. However, studies on the health of women prisoners are primarily conducted in Western societies, and studies in other countries are rare. Thailand, a non-western country, is no exception to this.

**Objectives:**

The objectives of this study were to assess the current levels of anxiety and depression among women drug offenders in Thailand and to identify possible associated factors.

**Methods:**

Data were collected from a sample consisting of 554 women drug offenders serving sentences of eight years or more. Stratified random sampling with proportionate stratification was employed during the data collection. The female inmates were being held in three categories of prisons: correctional institutions, central prisons, and provincial prisons. A single question was used to measure self-perceived levels of anxiety and depression: none, moderate, or substantial. Ordered logit regression was employed in the data analysis.

**Findings:**

One out of five (21.1%) of the inmates in the sample reported no perceived current anxiety and depression, 61.7% reported moderate anxiety and depression, and 17.1% reported having substantial perceived levels of anxiety and depression. It was found that chronic health conditions or disease, concerns about economic status, and feelings of shame were associated with the perceived anxiety and depression reported by the inmates.

**Conclusion:**

The study’s findings suggest that integrated mental health services that emphasize a holistic approach that acknowledges the intersectionality of women’s mental health and societal gender roles should be provided in prisons. Regular mental health screening and accessible mental health services are essential for all incarcerated women. Empowerment programs during confinement can boost self-esteem and thus lead to better post-release outcomes. The government should also implement programs to alleviate the financial burden on prisoners’ households.

## Introduction

In the period 2015–2020, the number of female prisoners exceeded 700,000 globally, with Thailand ranking either fourth or fifth in terms of the number of women incarcerated [1, 2]. In 2018, per capita female imprisonment in Thailand was the highest in the world at 140 per 100,000 [3]. A majority of these female prisoners (over 80%) were detained or sentenced under Thailand’s strictly enforced Illicit Drug Act, B.E. 2548 (2003) [4, 5]. Particularly since the declaration of the “war” on drugs in 2003, the enforcement of Thailand’s drug laws has led to a significant increase in the number of incarcerated individuals, especially women, on drug-related charges [2, 6–8]. Convictions can result in a wide range of sentences from minor penalties to life imprisonment or even the death penalty [9, 10].

As the number of prisoners has grown, the increasing prevalence of mental health problems among them has emerged as a significant public health concern [11–13]. Research conducted in Western countries has consistently demonstrated that incarcerated individuals experience higher rates of psychiatric disorders than the general population [13–16]. When the prevalence of depression among prisoners in developed and developing countries is compared, a significantly higher occurrence in developing countries can be found (39.2% versus 33.1%) [17]. In addition, female prisoners often experience more severe mental health symptoms than their male counterparts [13, 15, 16, 18–21].

Various factors contribute to the mental health of prisoners, including sociodemographic factors, sentence conditions, the prison environment, and external factors. Sociodemographic factors such as education level, employment, and drug addiction also play significant roles [22–30], as do trauma, feelings of shame, and physical health issues [31–41].

Long sentences and re-incarceration can lead to profound adverse effects on mental health [11, 25, 42]. Within the prison environment, factors such as stringent rules, punishment, bullying, lack of privacy, overcrowding, limited access to meaningful activities, and the availability or lack of mental health services have substantial impacts [43, 44].

Outside prison, family support plays a vital role in mental health, but incarceration often strains family relationships [29, 45, 46]. In addition, women often bear financial responsibilities for their families even before entering prison, and imprisonment can affect a household’s financial stability [8, 47, 48]. Factors such as stigmatization, frequency of visits, and the availability of communication channels also influence the mental health of prisoners [19, 41, 45].

Despite the prevalence of mental health issues among incarcerated women in Thailand, research in this area has been limited. Previous studies on anxiety and depression in incarcerated women have used small sample sizes and lacked adequate controls for confounding factors [49, 50]. This study aimed to fill this gap by assessing self-perceived levels of anxiety and depression and by considering associated factors among women prisoners serving long sentences for drug-related offenses in Thailand using a sufficient sample size for the application of inferential statistics.

The Department of Corrections in Thailand defines a long-term sentence as imprisonment exceeding four years; however, in this study, we considered sentences of eight years or longer as being long-term. This distinction was made because sentences of eight years or longer are often given to individuals convicted of drug offenses, including the possession, import, or export of dangerous drugs such as amphetamines [51].

Anxiety is often marked by pervasive emotions such as fear, apprehension, and unease, whereas depression manifests as profound sadness and a notable loss of interest [52]. In this study, anxiety and depression were considered together due to their recognized comorbidity [53, 54]. Thus, self-perceived anxiety and depression were evaluated based on a single question: “What is your level of anxiety and depression today?”

The results of this study will contribute to a deeper understanding of the mental health needs of incarcerated women and the importance of tailored mental health services for women inmates.

## Materials and methods

### Study design and setting

In this study, a cross-sectional design was employed. The survey began with a stratified random sampling of 15 prisons as part of the “Enhancing Lives of Female Inmates” project conducted by the Royal Inspire Project (Kamlungjai Project). The prisons were divided into three groups: correctional institutions, central prisons, and provincial prisons; one institution was randomly selected from each group. The selected prisons were a provincial prison in southern Thailand, a central prison in northeastern Thailand, and a correctional institution in the north of the country. Provincial prisons typically accommodate inmates serving sentences of up to 15 years, whereas central prisons are designated for those given sentences of up to 25 years. Correctional institutions house the largest population of inmates awaiting execution. All three selected prisons were considered to be gender-sensitive.

### Population and sampling

The survey sample was constructed by applying probability proportional to size to a list of all target inmates at the three selected prisons. This produced a randomly selected sample consisting of 576 women inmates: 442 from the correctional institution, 112 from the central prison, and 22 from the provincial prison. Of these, 127 inmates declined to participate in the study, and those on a reserve list replaced them. After some exclusions (due to, for example, non-responses for some variables), an eligible sample of 554 prisoners remained. These prisoners were all drug offenders who had been sentenced to imprisonment for periods of eight years to life or who had been given the death penalty.

Yamane’s simplified formula [55] was used to calculate the desired sample size with a 95% confidence interval and a precision level of 5%, which determines a conservative sample size with maximum variability (p = .5). The formula can be written as

$$n=\frac{N}{1+{Ne}^{2}},\;370=\frac{4,917}{1+{4,917*0.05}^{2}}$$

where, n = sample size, N = population size (4,917), and e = level of precision (0.05).

Applying this formula, gave a sample size of 370 from a list of 4,917 individuals who met the study criteria. During a pretest performed at the women’s central correctional institute in central Thailand, there was a 55% non-response rate. As the non-response rate is known to induce bias in the estimator [56], to compensate for an expected non-response rate of 55% (206 samples), the final sample size was set at 576.

### Data collection

The data were collected using an interviewer-administered questionnaire. A pretest of the questionnaire was conducted on a sample of 35 prisoners to assess both its validity and reliability. Face validity was used to assess the validity of the questions [56]. Following the pretest, the questions were revised to ensure that the prisoners in the sample would understand the meaning of the questions. The questionnaire was found to have a Cronbach’s alpha reliability (α) of 0.755.

The survey was conducted in November and December of 2015 through face-to-face interviews by trained interviewers. Each interview lasted 45–60 minutes. Data on inmates’ sociodemographic characteristics, family circumstances, experience of the criminal justice process, views on the court verdict, and perceived health were collected.

### Study variables

The outcome variable was perceived anxiety and depression; the survey answers were classified into three levels: no anxiety and depression, moderate anxiety and depression, and substantial anxiety and depression. The independent variables included inmates’ sociodemographic characteristics, family circumstances, and the details of their sentences.

Perceived anxiety and depression were the focus of this study because these are common significant health problems for women prisoners worldwide [15, 57, 58]. The survey employed a single question that combined anxiety and depression because anxiety and depression are mutually reinforcing and have previously been considered to be comorbid conditions [53, 54]. Previous studies have also found that a single question on anxiety and depression is essential in identifying potentially anxious and depressive people [59, 60].

### Data analysis

Both descriptive and multivariate analysis were applied to the data acquired by the survey. Descriptive analysis was used to understand the basic features of the sample. Multivariate ordered logistic regression analysis was used to identify factors associated with perceived current anxiety and depression and to estimate the odds ratios for these factors in relation to the outcomes. Moreover, variables that demonstrated statistically significant correlations (those with a p-value < .05) in a simple ordered logistic regression were incorporated into a multiple ordered logistic regression using the “enter” method to control for confounding factors [61].

A correlation matrix was used to perform a multicollinearity test on the results; the parallel line test of assumptions was also performed. The results showed that the methods used had not violated the assumptions made (see **S1** and **S2 Tables**). The goodness of fit of the ordered logit regression model was also tested for using Pearson’s chi-square statistic and deviance since both categorical and continuous covariates were permitted in the model [62]. It was found that Pearson’s chi-square and deviance statistics for the model were both greater than .05, indicating that the model used was appropriate and fit the data well [61] (**S3 Table**).

### Ethical considerations

Written and verbal consent was obtained from all respondents. Inmates who declined to participate in the study were not disadvantaged in any way. Respondents were compensated for their time with a payment of 100 THB (approximately 3 USD). The study was approved by the Institute for Population and Social Research Institutional Review Board, Mahidol University; the COA. No. of the study is 2015/1-1-136.

## Results

### Characteristics of respondents

Of the 554 women inmates surveyed, the majority were less than 40 years old. The largest proportion of respondents were Buddhists, representing slightly more than four-fifths of the sample. Most of the sample were Thai, married, and had completed primary school. A majority had also been gainfully employed prior to being imprisoned. Somewhat less than half of the sample reported that they had a chronic disease or condition (e.g., diabetes, hypertension, or chronic pain).

Most respondents had at least one child; nearly 30% of these children were under 11 years old. Only 11.6% of respondents reported that they had been addicted to drugs before their incarceration. In addition, almost two-thirds felt ashamed due to their arrest and imprisonment.

More than 50% of the incarcerated women were the person in their household who was mainly responsible for paying family debts, and one-fourth were the primary breadwinners. In relation to their concerns about parenting, almost 25% were worried about their children’s negative behavior, while nearly 50% were concerned about their household’s economic hardship in their absence.

Most respondents had no history of imprisonment, suggesting that they were first-time offenders. However, almost one-tenth reported recidivism. Slightly more than half of the sample had received a sentence of 20 to 30 years, and one-fifth had been sentenced to life imprisonment. There were only nine cases (1.6%) where the respondent had been sentenced to death. The majority of respondents had been incarcerated for less than one year and still had 10 to less than 25 years of their sentences left to serve. The majority (84.1%) felt they had not been given a sentence commensurate with their drug offense (indicating a lack of proportionality in the punishment). Slightly more than half completely accepted the legal penalties they had suffered and perceived that the severity of their sentences was equal to the gravity of their drug offense (Table 1).

**Table 1:** Sample characteristics (n = 554).

Characteristics	N	%
**Sociodemographic characteristics**		
**Age (years)**		
Under 30	101	18.2
30–39	203	36.6
40–49	129	23.3
50 or over	121	21.8
**Religion**		
Buddhist	471	85.0
Other	83	15.0
**Ethnicity**		
Thai	352	63.5
Ethnic groups in Thailand	133	24.0
Non-Thai	69	12.5
**Marital status**		
Single	71	12.8
Married	330	59.6
Widowed/divorced/separated	153	27.6
**Education**		
Primary school	401	72.4
Secondary school	153	27.6
**Employment status before imprisonment**		
Unemployed	81	14.6
Employed	473	85.4
**Has a chronic disease or condition**		
No	294	53.1
Yes	260	46.9
**Has children aged under 11 years**		
No	392	70.8
Yes	162	29.2
**Drug-addicted before imprisonment**		
No	490	88.4
Yes	64	11.6
**Feels ashamed of being imprisoned**		
No	203	36.6
Yes	351	63.4
**Family circumstances**		
**Main person responsible for family’s debts before imprisonment**		
No	253	45.7
Yes	301	54.3
**Main family breadwinner before imprisonment**		
No	419	75.6
Yes	135	24.4
**Concerned about children’s behavior (aggressive/depressive/isolative behavior)**		
No	420	75.8
Yes	134	24.2
**Concerned about worsening of household’s economic status or having more debt**		
No	287	51.8
Yes	267	48.2
**Sentence conditions**		
**Previous imprisonment**		
No	505	91.2
Yes	49	8.8
**Term of sentence**		
Less than 20 years	60	10.8
20 to 30 years	297	53.6
More than 30 years	84	15.2
Life imprisonment	104	18.8
Death penalty	9	1.6
**Length of sentence already served**		
Less than 6 months	171	30.9
6 months to less than 1 year	132	23.8
1 year to < 2 years	90	16.2
2 years to < 5 years	74	13.4
5 years or more	87	15.7
**Length of sentence remaining**		
Less than 10 years	127	22.9
10 to < 25 years	296	53.4
25 years or more	76	13.7
Life imprisonment	47	8.5
Death penalty	8	1.4
**Acceptance of criminal charge/penalty**		
Completely accepts	283	51.1
Accepts to some extent	110	19.9
Does not accept at all	161	29.1
**Opinion about proportionality of received penalty**		
Not proportional	466	84.1
Proportional	88	15.9
**Opinion about fairness of received penalty**		
Perceived as unfair	100	18.1
Perceived as fair	454	81.9

### Perceived current levels of anxiety and depression

**Fig 1:**
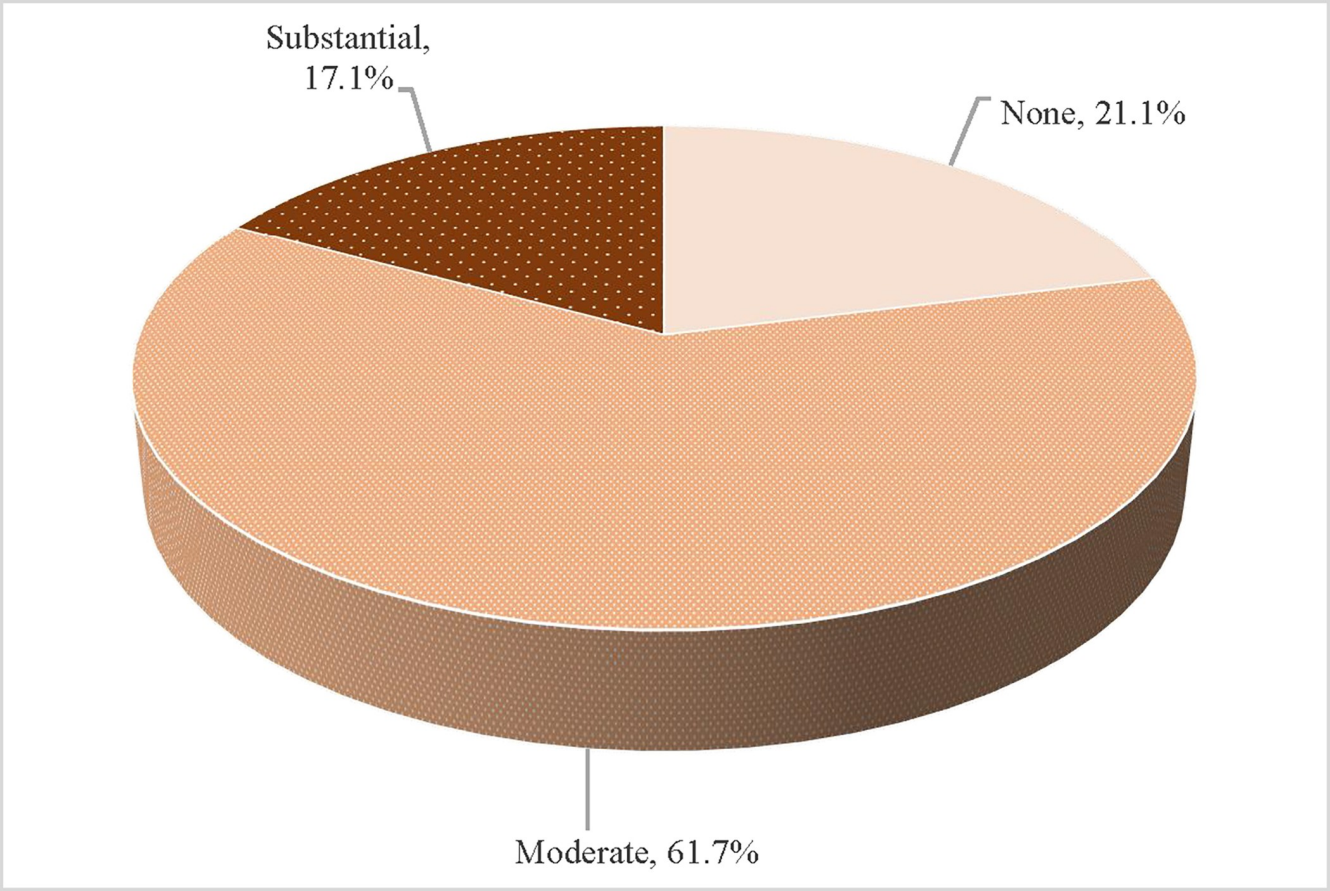
Levels of perceived current anxiety and depression among long-term prison inmates in Thailand.

The perceived current levels of anxiety and depression reported by the respondents are shown in **Fig 1**. Only slightly over one-fifth (21.1%) of the sample reported no perceived anxiety or depression. Over three-fifths (61.7%) reported moderate anxiety and depression, and less than one-fifth (17.1%) reported they were suffering from substantial anxiety and depression. Overall, the reported current prevalence of anxiety and depression among the imprisoned women was 78.8%. The levels of perceived anxiety and depression analyzed according to the sociodemographic characteristics of the women inmates, concerns about family, and sentencing conditions are shown in **S4 Table**.

### Factors associated with perceived current levels of anxiety and depression among women inmates

Table 2 presents the results of the ordered logistic regression, including the odds ratios; these results are statistically significant at the 95% confidential interval. The model fits the data, explaining how the model provides good predictions. The log-likelihood has a value of 245.718, the LR chi-square value is 57.033, with significant probability > chi-square (p < .05). The value of Nagelkerke’s R^2^ suggests that the model can account for 11.6% of the variance in the tier of entry. In terms of sociodemographic factors, the results show that women inmates with a chronic condition or disease were 1.6 times more likely to report moderate or substantial levels of anxiety and depression than those who did not have a chronic condition or disease. Feelings of shame due to conviction were also connected with perceived anxiety and depression. Respondents with feelings of shame were almost 1.9 times more likely to report moderate or substantial anxiety and depression compared to those without these feelings.

**Table 2:** Ordered logistic regression model for estimating factors associated with perceived current levels of anxiety and depression among women inmates (n = 554).

Characteristics	Adjusted OR	95% confidence interval	
Sociodemographic characteristics		Lower bound	Upper bound
**Religion**			
Other (reference)			
Buddhist	0.687	−.856	.105
**Has a chronic disease or condition**			
No (reference)			
Yes	1.614*	.137	.821
**Feels ashamed of being imprisoned**			
No (reference)			
Yes	1.881*	.269	.995
**Family circumstances**			
**Main family breadwinner before imprisonment**			
Yes (reference)			
No	1.192	−.226	.578
**Concerned about children’s negative behavior (aggressive/depressive/isolative behavior)**			
No (reference)			
Yes	0.681	−.790	.022
**Concerned about worsening of household’s economic status or having more debt**			
No (reference)			
Yes	2.063**	.366	1.083
**Log-likelihood**		**245.718**	
**LR χ** ^**2**^		**57.033**	
**Probability > χ** ^**2**^		**0.000**	
**Pseudo Nagelkerke R** ^**2**^		**.116**	

Note: the dependent variable measures perceived anxiety and depression (based on an ordinal scale with 0 = none, 1 = moderate, and 2 = substantial).

** P < .001

* P < .05

The analysis also shows that family dependency, especially in households with economic difficulties, influenced the women’s perceived levels of anxiety and depression. Incarcerated women concerned about the deterioration of their household’s economic status (i.e., their ability to pay their debts) were twice as likely to report moderate or substantial anxiety and depression than those who did not have such concerns. It is notable that the sentence conditions did not explain the inmates’ perceived levels of anxiety and depression.

## Discussion

This study was concerned with the mental health of women in Thailand who had been incarcerated for drug offenses and who had been sentenced to periods of imprisonment ranging from eight years to life or to death. Current self-perceived levels of anxiety and depression among the inmates were recorded. It was found that 78.8% of respondents reported moderate and substantial levels of anxiety and depression. Statistical analysis revealed three significant factors associated with higher levels of anxiety and depression. These were: 1) a chronic health condition, 2) feelings of shame, and 3) concern about the worsening economic conditions of the inmate’s family. It was also observed that the sentence conditions did not help explain the levels of anxiety and depression experienced by the women studied. A possible reason for this lack of significance may be that all of the respondents were serving long-term sentences, meaning that, in this aspect, there was little variation within the sample.

According to the results, the prevalence of perceived anxiety and depression among imprisoned women in Thailand is considerably higher than that in the general population (78.8% versus 40%) [63]. This finding is consistent with the results of previous studies, which have indicated that the incarcerated population suffers from higher rates of mental health problems than the general population [16–20]. However, the prevalence of anxiety and depression found in this study was actually higher than that found in previous studies of Thai women prisoners: one study [50] found that the percentage of women prisoners with mental health problems was 48%; another [49] found a prevalence of 72%. Even though these different studies employed different tools to measure mental health, the prevalence of mental health-related problems among women inmates is undoubtedly higher than that among the general population. The findings of this study are, therefore, consistent with previous research and indicate that anxiety and depression are pervasive mental health problems among women prisoners [15, 58].

Incarceration is stressful since individuals are exposed to both acute and chronic stressors as a result of the harsh environment in which they are held [64]. Previous studies have indicated that, within the prison environment, various factors such as stringent rules, punishment, bullying, lack of privacy, overcrowding, and the limited availability of meaningful activities impact the mental health of prisoners [43, 44]. In Thailand, prisoners also experience both overcrowding and prolonged isolation as prisons often exceed their capacity [65]. As a result, inmates typically spend up to 14 hours a day in their prison cells [66], adding to the stress of confinement. They must also endure unkind treatment from prison staff and other inmates [65]. Another study conducted in Thailand revealed that the impact of imprisonment on the mental health of women in Thailand is exacerbated by gender-insensitive conditions within correctional facilities [47].

The unacceptably high prevalence of anxiety and depression among incarcerated women found in this study shows that the provision of mental healthcare services for prisoners is essential. Although the Thai Ministry of Public Health provides standard counseling services to prisoners once a month [67], this may not be sufficient in many cases. Enhanced training programs for correctional officers are essential so that they can better support women in criminal justice settings. These programs should be tailored to reflect an understanding of the unique challenges that imprisoned women face and address their specific needs; the programs should also include counseling training [68].

Providing counseling for inmates who have recovered from mental health problems is another strategy that can be used to improve access to essential counseling services for inmates with anxiety and depression. Appropriate mental health awareness training for all prison staff is also necessary. Such programs help provide incarcerated women with prison staff or other inmates to talk to; these can function as peer networks or mentors with limited resources [69, 70]. In addition, mental health services provided for women inmates must be based on the principles of the “Bangkok Rules” and adopt a gender-sensitive approach [8]. Thailand took a significant step toward addressing mental health issues within its prison system by implementing “Happy Centers” in selected pilot prisons in 2015 in line with the Bangkok Rules. The primary objective of this initiative is to facilitate trauma recovery and provide essential psychological support [71]. This program is a viable alternative approach to mitigating anxiety and depression among prisoners.

Similar to results documented elsewhere [37], the results of the study suggest a relationship between physical and mental health. Women inmates suffering from a chronic disease or with a chronic condition reported a significantly higher level of anxiety and depression than the other inmates. This indicates that some chronic diseases and conditions lead to emotional stress; this can trigger mental disorders in both the general and incarcerated population [34, 72]. In addition, previous study on Aboriginal women has indicated that physical and mental deterioration in women can result from social and economic constraints. Coupled with caregiving responsibilities, these constraints frequently lead women to neglect their own health [73]. In Thai society, in their roles as daughters, wives, and mothers, women often prioritize the needs of others, sometimes neglecting to care for themselves [74]. The results presented here reinforce the importance of providing adequate mental health services in prisons for those who need them, in particular for women. Every prisoner who needs help with mental health should be treated individually.

Feelings of shame play a significant role in the development of anxiety among incarcerated women. Women’s feelings of shame are often related to the stigma of incarceration [75], and this stigma plays a significant role in the development of anxiety among incarcerated women [76]. As a result of gender-related stereotypes, imprisoned women feel more ashamed and endure more societal blame than male prisoners do [26, 75]. Thai women who have committed a crime and been imprisoned are often regarded as “fallen” because imprisonment leads to them being branded as failures in terms of upholding the feminine ideals of being a virtuous woman, a responsible mother, or a filial daughter [77]. The results of this study confirm that feelings of shame are significantly correlated with perceived anxiety and depression [36], and imprisonment leads to societal condemnation and feelings of shame [78]. In Thailand, individuals who have experienced incarceration often face stigmatization [79]. Moreover, studies elsewhere show that, when people respond to shame, this can result in self-stigmatization, leading to feelings of hopelessness and isolation [80, 81]. Therefore, existing empowerment programs that increase self-esteem for incarcerated women should be enhanced [82].

Many women end up in prison due to the link between poverty and drug-related crime. Poverty often forces women into criminal activities [83], and even while incarcerated, they continue to be concerned about the economic well-being of their families [84]. Economic hardship is a source of distress [22] and can lead to anxiety and isolation [28]. Women prisoners often face financial pressures due to family responsibilities, which significantly affects their mental well-being [24, 29, 85]. Nearly 50% of the respondents in the survey expressed concerns about their family’s financial situation, highlighting the connection between incarcerated women’s anxiety and depression and their worries about their families’ possibly worsening economic conditions. This observation aligns with previous research, which has demonstrated that economic hardship has an adverse impact on the mental health of incarcerated individuals, leading to increased depression and anxiety levels [86]. In addition, previous studies have pointed out that female Thai drug offenders frequently experience economic marginalization and bear the primary responsibility for supporting their families financially [87–89].

According to a report from the Thailand Institute of Justice, women prisoners incur increased debts due to legal costs (including lawyer fees, court fees, execution officer fees, fees for the examination outside the court, and litigation costs) as well as penalties incurred during their court case [90]. Over 80% of the women in this study were the primary income earners for their families prior to their imprisonment. It is known that families commonly face financial pressures when the primary breadwinner is incarcerated [29]. This finding is consistent with a previous study in Thailand, which indicated that over 75% of women drug offenders are of working age and serve as the primary income providers for their families [45]. Furthermore, a separate study highlighted that women often bear the responsibility of providing financial support to their families before entering prison [91].

This study also found that 54.8% of imprisoned women were responsible for paying their household debt. In Thai society, there is a prevalent expectation that women should shoulder the family’s financial responsibilities while also fulfilling their roles as mothers, wives, and daughters [77, 92]. Hence, an inability to fulfil family obligations can increase imprisoned women’s worries and lead to self-perceived depression and anxiety [92].

Although the Department of Corrections in Thailand provides work programs for women in prison, the payment that prisoners receive is lower than the minimum wage [93]. The findings of this study suggest that there is a need to improve the wages paid under existing prison work programs. This is critical for mitigating the economic hardship experienced by women inmates and their families due to loss of income. Engaging in income-generating programs during incarceration can help individuals increase their ability to cover their legal expenses and alleviate the financial burden on their families. Participation in these programs may reduce depression related to economic pressures [94, 95].

This study has certain limitations. In the survey, anxiety and depression were combined into a single question; the study also relied on self-reported data to determine the prevalence of these conditions. However, despite these limitations, the acquired data remain valuable as an initial tool for identifying women who may genuinely require mental health care. If the approach used in this study is to be adopted more widely, it should undergo further validation before being applied. In addition, further studies that focus on the clinical assessment of anxiety and depression as separate variables are needed. Caution is needed when making generalizations based on the results of this study because of the limited number of prisons that were the focus of the Royal Inspire Project. The Royal Inspire Project aims to improve the lives of women inmates but does not represent the entirety of the country’s female prison population. In addition, this survey focused only on women inmates serving sentences of eight years or more.

Despite these limitations, the results of this study provide a more systematic overview of the mental health of women prisoners in Thailand than has been provided by previous studies. The sample size that was used allowed the factors that influence women prisoners’ mental health to be investigated and for the control of confounding factors.

## Conclusions

Based on the results of this study, it is recommended to incorporate mental health services within correctional facilities, with a focus on adherence to the gender-sensitive guidelines outlined in the “Bangkok Rules”. It is crucial that regular mental health screening is conducted and to ensure accessible services for all incarcerated women. Empowerment programs offered during incarceration can enhance self-esteem and lead to more favorable outcomes upon release. The government should also initiate programs aimed at alleviating the financial hardships faced by prisoners’ families.

## Supporting information

S1 Table Multicollinearity test. (DOCX)

S2 Table Parallel line test assumptions. (DOCX)

S3 Table Goodness of fit the test. (DOCX)

S4 Table Percentage distribution of level of perceived current anxiety and depression by sample*’*s characteristics. (DOCX)
