# Supplementary material for: Perceived anxiety and depression and associated factors among women inmates with a long-term sentence in Thailand
Source: PLoS One. 2024 Mar 1;19(3):e0299318. doi: 10.1371/journal.pone.0299318 (PMC10906842; doi:10.1371/journal.pone.0299318)
Supplement: S1 Table — (DOCX) [file pone.0299318.s001.docx]

**S1 Table: Results of multicollinearity test by using the correlation matrix**

|  | **V1** | **V2** | **V3** | **V4** | **V5** | **V6** |
| --- | --- | --- | --- | --- | --- | --- |
| **V1** | 1.000 |  |  |  |  |  |
| **V2** | -.041 | 1.000 |  |  |  |  |
| **V3** | -.088^*^ | .025 | 1.000 |  |  |  |
| **V4** | -.132^**^ | .054 | .048 | 1.000 |  |  |
| **V5** | .094^*^ | -.009 | -.150^**^ | -.105^*^ | 1.000 |  |
| **V6** | -.101^*^ | .019 | .141^**^ | .169^**^ | -.155^**^ | 1.000 |

Remark: V1= Religion, V2 = Has a chronic disease or condition, V3 = Feels ashamed of being imprisoned V4 = Main family breadwinner before imprisonment, V5 = Concerned about children's behavior, V6 = Concerned about worsening of household’s economic status or having more debt

** P< .001; and *P< .05
