# Supplementary material for: Perceived anxiety and depression and associated factors among women inmates with a long-term sentence in Thailand
Source: PLoS One. 2024 Mar 1;19(3):e0299318. doi: 10.1371/journal.pone.0299318 (PMC10906842; doi:10.1371/journal.pone.0299318)
Supplement: S2 Table — (DOCX) [file pone.0299318.s002.docx]

**S2 Table: Test of Parallel Lines^a^**

|  | | | | |
| --- | --- | --- | --- | --- |
|  | | | | |
| **Model** | **-2 Log Likelihood** | **Chi-Square** | **df** | **Sig.** |
| Null Hypothesis | 245.718 |  |  |  |
| General | 237.168 | 8.551 | 6 | .200 |
| The null hypothesis states that the location parameters (slope coefficients) are the same across response categories.  a. Link function: Logit. | | | | |
