# Supplementary material for: Perceived anxiety and depression and associated factors among women inmates with a long-term sentence in Thailand
Source: PLoS One. 2024 Mar 1;19(3):e0299318. doi: 10.1371/journal.pone.0299318 (PMC10906842; doi:10.1371/journal.pone.0299318)
Supplement: S3 Table — (DOCX) [file pone.0299318.s003.docx]

| **S3 Table. Goodness-of-Fit** | | | |
| --- | --- | --- | --- |
|  | Chi-Square | df | Sig. |
| Pearson | 80.182 | 94 | .844 |
| Deviance | 94.763 | 94 | .459 |
| Link function: Logit. | | | |
