# Supplementary material for: Perceived anxiety and depression and associated factors among women inmates with a long-term sentence in Thailand
Source: PLoS One. 2024 Mar 1;19(3):e0299318. doi: 10.1371/journal.pone.0299318 (PMC10906842; doi:10.1371/journal.pone.0299318)
Supplement: S4 Table — (DOCX) [file pone.0299318.s004.docx]

**S4 Table. Percentage distribution of perceived current levels of anxiety and depression by sample’s characteristics** **(n=554)**

| **Characteristics** | **Perceived current levels of anxiety and depression** | | | **Total** | |
| --- | --- | --- | --- | --- | --- |
|  | **None** | **Moderate** | **Substantial** | **N** | **%** |
| **Sociodemographic characteristics** |  |  |  |  |  |
| **Age (years)** |  |  |  |  |  |
| Under 30 | 22.8 | 64.4 | 12.9 | 101 | 18.2 |
| 30-39 | 16.7 | 66.5 | 16.7 | 203 | 36.6 |
| 40-49 | 23.3 | 58.1 | 18.6 | 129 | 23.3 |
| 50 or over | 24.8 | 55.4 | 19.8 | 121 | 21.8 |
| **Religion** |  |  |  |  |  |
| Buddhist | 22.3 | 62.2 | 15.5 | 471 | 85.0 |
| Other | 14.5 | 59.0 | 26.5 | 83 | 15.0 |
| **Ethnicity** |  |  |  |  |  |
| Thai | 23.0 | 60.8 | 16.2 | 352 | 63.5 |
| Ethnic groups in Thailand | 18.0 | 60.9 | 21.1 | 133 | 24.0 |
| Non-Thai | 17.4 | 68.1 | 14.5 | 69 | 12.5 |
| **Marital status** |  |  |  |  |  |
| Single | 25.4 | 64.8 | 9.9 | 71 | 12.8 |
| Married | 19.4 | 63.3 | 17.3 | 330 | 59.6 |
| Widowed/divorced/separated | 22.9 | 56.9 | 20.3 | 153 | 27.6 |
| **Education** |  |  |  |  |  |
| Primary school | 21.7 | 60.3 | 18.0 | 401 | 72.4 |
| Secondary school | 19.6 | 65.4 | 15.0 | 153 | 27.6 |
| **Employment status before imprisonment** |  |  |  |  |  |
| Unemployed | 27.2 | 60.5 | 12.3 | 81 | 14.6 |
| Employed | 20.1 | 61.9 | 18.0 | 473 | 85.4 |
| **Has a chronic disease or condition** |  |  |  |  |  |
| No | 23.5 | 64.3 | 12.2 | 294 | 53.1 |
| Yes | 18.5 | 58.8 | 22.7 | 260 | 46.9 |
| **Has children aged under 11 years** |  |  |  |  |  |
| No | 21.7 | 61.2 | 17.1 | 392 | 70.8 |
| Yes | 19.8 | 63.0 | 17.3 | 162 | 29.2 |
| **Drug-addicted before imprisonment** |  |  |  |  |  |
| No | 20.8 | 62.0 | 17.1 | 490 | 88.4 |
| Yes | 23.4 | 59.4 | 17.2 | 64 | 11.6 |
| **Feels ashamed of being imprisoned** |  |  |  |  | 48.2 |
| No | 30.0 | 59.1 | 10.8 | 203 | 36.6 |
| Yes | 16.0 | 63.2 | 20.8 | 351 | 63.4 |
| **Family circumstances** |  |  |  |  |  |
| **Main person responsible for family's debts before imprisonment** |  |  |  |  |  |
| No | 19.0 | 62.8 | 18.2 | 253 | 45.7 |
| Yes | 22.9 | 60.8 | 16.3 | 301 | 54.3 |
| **Main family breadwinner before imprisonment** |  |  |  |  |  |
| No | 18.9 | 63.0 | 18.1 | 419 | 75.6 |
| Yes | 28.1 | 57.8 | 14.1 | 135 | 24.4 |
| **Concerned about children's behavior (aggressive/depressive/isolative behavior)** |  |  |  |  |  |
| No | 24.8 | 59.3 | 16.0 | 420 | 75.8 |
| Yes | 9.7 | 69.4 | 20.9 | 134 | 24.2 |
| **Concerned about worsening of household’s economic status or having more debt** |  |  |  |  |  |
| No | 28.9 | 58.9 | 12.2 | 287 | 51.8 |
| Yes | 12.7 | 64.8 | 22.5 | 267 | 48.2 |
| **Sentence conditions** |  |  |  |  |  |
| **Previous imprisonment** |  |  |  |  |  |
| No | 21.8 | 61.8 | 16.4 | 505 | 91.2 |
| Yes | 14.3 | 61.2 | 24.5 | 49 | 8.8 |
| **Term of sentence** |  |  |  |  |  |
| Less than 20 years | 15.0 | 68.3 | 16.7 | 60 | 10.8 |
| 20 thru 30 years | 21.5 | 63.0 | 15.5 | 297 | 53.6 |
| Over 30 years | 23.8 | 57.1 | 19.0 | 84 | 15.2 |
| Life imprisonment | 20.2 | 59.6 | 20.2 | 104 | 18.8 |
| Death penalty | 33.3 | 44.4 | 22.2 | 9 | 1.6 |
| **Length of sentence already served** |  |  |  |  |  |
| Less than 6 months | 23.4 | 55.6 | 21.1 | 171 | 30.9 |
| 6 months - less than 1 year | 16.7 | 65.2 | 18.2 | 132 | 23.8 |
| 1 year to < 2 years | 23.3 | 66.7 | 10.0 | 90 | 16.2 |
| 2 years to < 5 years | 21.6 | 64.9 | 13.5 | 74 | 13.4 |
| 5 years or more | 20.7 | 60.9 | 18.4 | 87 | 15.7 |
| **Length of sentence remaining** |  |  |  |  |  |
| Less than 10 years | 22.0 | 66.9 | 11.0 | 127 | 22.9 |
| 10 to < 25 years | 20.3 | 61.8 | 17.9 | 296 | 53.4 |
| 25 years or more | 23.7 | 52.6 | 23.7 | 76 | 13.7 |
| Life imprisonment | 23.4 | 59.6 | 17.0 | 47 | 8.5 |
| Death penalty | 0.0 | 75.0 | 25.0 | 8 | 1.4 |
| **Acceptance of criminal charge /penalty** |  |  |  |  |  |
| Completely accepts | 20.1 | 63.3 | 16.6 | 283 | 51.1 |
| Accepts to some extent | 23.6 | 53.6 | 22.7 | 110 | 19.9 |
| Does not accept at all | 21.1 | 64.6 | 14.3 | 161 | 29.1 |
| **Opinion about proportionality of received penalty** |  |  |  |  |  |
| Not proportional | 20.2 | 62.2 | 17.6 | 466 | 84.1 |
| Proportional | 26.1 | 59.1 | 14.8 | 88 | 15.9 |
| **Opinion about fairness of received penalty** |  |  |  |  |  |
| Perceived as unfair | 19.8 | 63.0 | 17.2 | 454 | 18.1 |
| Perceived as fair | 27.0 | 56.0 | 17.0 | 100 | 81.9 |
